# Effectiveness of School-Based Interventions for Preventing Obesity in Children: A Narrative Review

**DOI:** 10.7759/cureus.75104

**Published:** 2024-12-04

**Authors:** Abdullah Alhelal, Maedah Salah AlSalem, Faysal Mohammed A Alasmari, Shatha Abdulrahman Alqarni, Rahaf Mohammed A Alamri, Rahaf Abdullah Ali Alshahrani, Alrashdi Afnan Hassan M., Abdulwahab Almalki, Wajd Hussein D Alenezi, Noor Mohammed F Abdulrahim, Alsofri Ohood Aiyed B.

**Affiliations:** 1 Family Medicine, King Fahad Medical City, Riyadh, SAU; 2 Family Medicine, Vision Colleges, Riyadh, SAU; 3 General Medicine, Khamis Mushait General Hospital, Asir, SAU; 4 General Medicine, Al-Shariaa 2 Primary Health Care, Makkah, SAU; 5 General Medicine, King Abdulaziz University, Jeddah, SAU; 6 Family Medicine, King Fahad University Hospital, Alkhobar, SAU; 7 General Medicine, Maternity and Children Hospital, Dammam, SAU; 8 General Physician, King Fahad Hospital, Madinah , SAU; 9 General Medicine, Aldahiah Primary Health Care, The Northern Borders Health Cluster, Arar, SAU; 10 Family Medicine, Batterjee Medical College, Jeddah, SAU; 11 General Medicine, Jeddah Eye Hospital, Jeddah, SAU

**Keywords:** children, factors influencing effectiveness, nutritional education programs, obesity, physical activity programs, school-based interventions

## Abstract

The global rise in overweight and obesity among children presents significant health challenges. Schools, as key social environments, can effectively influence children's dietary and physical activity habits due to the substantial time students spend there. School-based interventions can reach nearly all children and impact their environment's sociocultural and policy aspects to promote healthier habits. This literature review summarizes the literature discussing the effectiveness of school-based interventions in preventing childhood obesity, identifies the most effective strategies, and provides evidence-based recommendations for future interventions. In August 2024, a comprehensive search was performed across PubMed, Scopus, and Web of Science to identify the key studies discussing our topic. We found that school-based interventions that integrate nutritional education and physical activity programs effectively reduce body mass index (BMI) and improve weight status among children. Extensive research and systematic reviews have shown significant reductions in BMI through these comprehensive approaches. Incorporating nutritional education into the school curriculum, with practical lessons and healthy food promotions, positively impacts dietary behaviors. Physical activity programs, such as regular physical education classes and extracurricular sports, are vital for promoting healthy weight status. Parental involvement further enhances these interventions, leading to better outcomes in weight status and energy balance-related behaviors. Policies that promote healthy eating and physical activity within schools, including restricting unhealthy food access and improving physical activity facilities, also reduce BMI and obesity risk among children.

## Introduction and background

The prevalence of overweight and obesity among children is rising globally, posing significant health challenges [1]. About 19.7% of U.S. children and adolescents, or about 14.7 million youths aged 2-19 years, were classified as obese from 2017 to March 2020 [2]. Childhood obesity is a risk factor for obesity in adulthood and is associated with various physical, social, and psychological issues. These include a heightened risk of noncommunicable diseases, social stigmatization, school bullying, and cyberbullying, leading to feelings of sadness and loneliness, engagement in high-risk behaviors, impaired students' self-image, low self-esteem, and negative stereotyping [3,4]. Childhood obesity is linked to other numerous health risks, including being the primary cause of pediatric hypertension. It is also associated with complications that typically develop later in life such as type 2 diabetes, orthopedic issues, a higher likelihood of coronary heart disease, and increased stress on weight-bearing joints [5,6]. 

Addressing obesity requires urgent action grounded in the best available evidence to ensure optimal outcomes. Current research encompasses interventions in various settings, including clinics, homes, community centers, and schools. Schools, in particular, stand out as effective venues for interventions due to their unique characteristics [7]. Schools provide a vital social environment for children, inherently exposing them to dietary and physical activity (PA) influences. Given that students spend a substantial portion of their lives in schools, school-based interventions can quickly reach nearly all children. Additionally, these interventions can impact the socio-cultural and policy aspects of the surrounding environment, fostering improved nutritional and PA habits [1,8,9].

Eating habits in schools are influenced by several factors, such as the availability of food and beverages from vending machines and school shops, along with offering nutrition education classes. Additionally, schools can encourage PA through structured programs and mandatory sports education, with many children also walking to and from school on weekdays [10]. Therefore, schools are well-positioned to foster both healthy nutrition and PA education [9]. 

This literature review critically summarizes existing research on the effectiveness of school-based interventions in preventing childhood obesity, while also identifying the most effective strategies and providing evidence-based recommendations for future interventions.

## Review

Methodology

In August 2024, a comprehensive search was performed across PubMed, Scopus, and Web of Science using the keywords: “School-Based Intervention,” “Obesity,” “Prevention,” and “Children,” and Medical Subject Headings (MeSH) to obtain the most recent and imported published article related to the aim of this study. Key references were identified through comprehensive discussions between the authors. The authors applied no filters or limitations during the search process except when we focused on the recently published studies (between 2014 and 2024).

Types of school-based interventions

The World Health Organization’s (WHO) commission on ending childhood obesity has acknowledged the crucial role of schools, offering six recommendations for policymakers globally. One key recommendation focuses on how schools can help reduce youth obesity by implementing comprehensive programs that foster healthy school environments and enhance health and nutrition literacy, and PA among school-age children [11]. Additionally, individual countries have recognized the importance of schools in improving child health. For instance, the U.S. Centers for Disease Control and Prevention highlights schools' significant role in promoting health among children and adolescents. This model emphasizes that health behaviors are more easily and effectively modified during youth, making schools an ideal setting for fostering lifelong healthy habits [12].

The primary goal of intervention programs aimed at preventing childhood obesity is to enhance nutrition and PA among the youngest children, as the likelihood of failure and relapse in established obesity is high [13]. These programs should be characterized by a wide-ranging scope, cost-effective policies, a social perspective, and a multi-sectoral and multi-level approach [14].

Nutritional Education Programs

Nutritional education programs in schools are designed to improve children’s dietary habits by providing them with the knowledge and skills necessary to make healthier food choices. Recent studies have highlighted various methods and approaches these programs use to maximize their effectiveness.

One common method is the integration of nutrition education into the school curriculum. This approach involves incorporating lessons on healthy eating into subjects such as science, health, and physical education (PE). For example, a study by Murimi et al. found that successful nutrition education interventions often included age-appropriate activities and were integrated into the broader curriculum to reinforce healthy eating messages [15]. These programs generally address topics like the significance of consuming fruits and vegetables, interpreting food labels, and the advantages of maintaining a balanced diet. Another effective method is the use of interactive and hands-on activities. Programs that engage students through cooking classes, gardening projects, and taste tests are particularly effective. These activities not only make learning about nutrition fun but also provide practical skills that students can use at home [16].

Parental involvement is also a key component of successful nutritional education programs. Engaging parents through workshops, newsletters, and take-home activities helps to reinforce the messages taught at school and encourages healthy eating habits at home. Interventions involving parents were more likely to achieve their objectives [15]. In addition, starting programs before lifelong habits are formed and peer pressure starts can be incorporated with parental administration and oversight.

Additionally, gaming programs developed for age-specific engagement would benefit these individuals. Digital tools such as apps, online games, and virtual lessons can make nutrition education more accessible and engaging for students. These tools can provide personalized feedback and track progress, making it easier for students to understand and apply what they have learned [15].

PA Programs

PA programs in schools are essential for promoting physical health and preventing obesity among children. These programs aim to increase students’ PA levels through structured exercises, sports, and active play. Recent studies have highlighted various methods and approaches these programs use.

One common method is the incorporation of regular PE classes into the school curriculum. These classes offer students the chance to participate in a variety of PAs, including running, jumping, and team sports. Regular PE classes significantly improve physical fitness and reduce obesity rates among students. These classes are often designed to be inclusive, ensuring that all students, regardless of their physical abilities, can participate and benefit [17].

Another effective approach is the implementation of extracurricular sports programs. These programs offer students additional opportunities to engage in PAs outside of regular school hours. They can include a wide range of activities, such as soccer, basketball, swimming, and dance [18].

Active transportation initiatives are another method used to promote PA. These initiatives encourage students to walk or cycle to and from school, thereby increasing their daily PA levels. Schools can support these initiatives by providing safe walking and cycling routes and organizing walking school buses [19].

Additionally, some schools implement comprehensive school-wide PA programs that integrate various activities throughout the school day. These programs can include short activity breaks during classes, active recess periods, and school-wide events such as sports days and fitness challenges [20].

Combined Interventions (Nutrition and PA)

Combined interventions that integrate both nutrition education and PA components are particularly effective in addressing childhood obesity. These interventions leverage the synergistic effects of promoting healthy eating habits alongside regular PA, resulting in more comprehensive health benefits for children.

One method of combined interventions involves structured programs that include classroom-based nutrition education paired with scheduled PA sessions. For instance, a study by Dinas demonstrated that such combined interventions significantly reduce body mass index (BMI), body weight, and fat mass, while improving overall physical health and quality of life. These programs often include lessons on healthy eating, cooking demonstrations, and PAs such as sports and exercise routines [21].

Another approach is the implementation of school-wide policies that promote both nutrition and PA. This can include providing healthier food options in school cafeterias and vending machines, along with ensuring that students have ample opportunities for PA throughout the school day. A study by Keawtep et al. emphasized the importance of such policies in creating sustainable health-promoting environments [22].

Additionally, some programs incorporate parental involvement to reinforce the messages taught at school. Engaging parents through workshops and take-home activities helps to ensure that healthy behaviors are supported both at school and at home. This holistic approach has been shown to enhance the effectiveness of combined interventions [23].

Policy and Environmental Changes

Policy and environmental changes within schools are crucial for creating a supportive environment that promotes healthy behaviors among students. These changes can include implementing school-wide policies that encourage healthy eating and PA, as well as modifying the physical environment to make healthier choices more accessible.

One effective method is the introduction of policies that regulate the availability of food and beverages in schools. For example, replacing sugary drinks and snacks in vending machines with healthier options can significantly impact students’ dietary habits [24].

Another approach involves modifying the school environment to encourage PA. This can include providing safe and accessible spaces for PAs, such as playgrounds and sports facilities, and ensuring that these areas are well-maintained and inviting [20].

Additionally, schools can implement comprehensive programs that integrate both policy and environmental changes. These programs can include initiatives like school gardens, which provide students with hands-on opportunities to learn about nutrition and engage in PA. Such integrated approaches are particularly effective in promoting healthy behaviors [16]. By implementing school-wide policies and modifying the physical environment, schools can significantly impact students’ health behaviors and contribute to the prevention of childhood obesity.

Effectiveness of interventions

Impact on BMI and Weight Status

School-based interventions have been widely studied for their effectiveness in improving BMI and weight status among children. These interventions often combine nutritional education with PA programs to create a comprehensive approach to obesity prevention and management.

A systematic review conducted in 2021 examined the impact of school-based interventions on obesity-related behaviors and BMI in primary school children. The review analyzed 38 studies and found that the interventions significantly reduced BMI and BMI z-scores. The interventions typically involved a combination of increased PA, decreased sedentary behavior, and improved nutrition behavior [25].

Another study that included 172 studies (189,707 participants) concluded that interventions aimed at preventing obesity in children aged five to 11 years old, which modify dietary intake and PA levels, have a positive impact on BMI. The findings indicate that these interventions can effectively reduce BMI and BMI z-scores in the short, medium, and long term. The evidence also supports the implementation of comprehensive programs that combine both dietary and PA modifications to achieve significant improvements in children’s weight status [26].

The integration of nutritional education into the school curriculum is also effective. This can include lessons on healthy eating habits, cooking demonstrations, and the promotion of healthy food choices in school cafeterias. School-based nutritional programs that included these components were associated with significant reductions in BMI and improvements in dietary behaviors. These programs often emphasize the importance of consuming fruits, vegetables, and whole grains while reducing the intake of sugary drinks and snacks [27].

PA programs are another crucial component of school-based interventions. These programs may encompass regular PE classes, extracurricular sports activities, and initiatives promoting active transportation. The influence of these programs on children's BMI and weight status is significant, as the integration of PA with nutrition education has been shown to effectively reduce BMI. A meta-analysis of 46 studies which was conducted in 2022 revealed a significant effect of these interventions, resulting in an average weight loss of 1.42 kg and achieving a 5% reduction in body weight [28].

Parental involvement is also a key factor in the success of school-based interventions. Programs that engage parents through workshops, take-home activities, and regular communication have been shown to enhance the effectiveness of interventions, as shown in a review by van de Kolk et al., which found that childcare-based interventions with direct parental involvement led to favorable outcomes in weight status and energy balance-related behaviors [29].

Changes in Dietary Behaviors

Nutritional education programs are a cornerstone of school-based interventions. These programs aim to enhance students’ knowledge about healthy eating and encourage the adoption of healthier dietary habits. Interventions incorporating curriculum-based nutrition education significantly improved children’s fruit and vegetable intake and nutrition knowledge. These programs often include classroom lessons, interactive activities, and hands-on experiences such as cooking classes and gardening projects [30].

Changes in the food environment within schools also play a crucial role in shaping children’s dietary behaviors. Regulations that ensure healthy food options are available in school cafeterias and vending machines have been shown to positively impact students’ food choices. For example, O’Brien et al. highlighted that interventions focusing on improving the food environment, such as providing healthier meal options and limiting access to sugary snacks and beverages, led to better dietary outcomes among students [31].

Changes in PA Levels

School-based interventions have demonstrated significant effectiveness in enhancing PA levels among children. These interventions often incorporate various methods as mentioned before, including structured PE classes, active breaks during lessons, and extracurricular sports programs.

A systematic review by Kriemler et al. found that school-based PA programs significantly increased in-school PA levels. The review highlighted that multi-component interventions, which combine PE classes, active breaks, and extracurricular activities, were particularly effective. These programs not only increased overall PA but also improved physical fitness and reduced sedentary behavior [32].

Another study found that interventions incorporating short PA breaks during lessons significantly increased students’ daily PA levels and improved their attentiveness and academic performance [33].

Additionally, experiential learning interventions are effective in enhancing PA outcomes among school-aged children. However, additional research is needed to determine their effectiveness in children under the age of five [34].

Long-Term Sustainability of Interventions

The long-term sustainability of school-based interventions is crucial for maintaining their effectiveness in improving children’s health outcomes. Recent studies have highlighted various factors that contribute to the sustainability of these interventions, including ongoing support, integration into school policies, and community involvement.

The integration of health interventions into the school curriculum and policies is crucial for ensuring their long-term sustainability. This approach helps to institutionalize healthy behaviors and makes them a regular part of the school environment. For example, integrating nutrition education into the curriculum and establishing school-wide policies that promote healthy eating can create a lasting impact on students’ dietary behaviors [35].

Community involvement is another key factor in the sustainability of school-based interventions. Another review of 13 studies found that programs with strong community support, including parental involvement and partnerships with local health organizations, were more likely to be sustained over time. Engaging the community helps to reinforce the messages taught at school and provides additional resources and support for maintaining healthy behaviors [36]. Additionally, Integration of community support programs with the trifold approach (activity, diet, and mental well-being) shows benefits when appropriately utilized [36].

Additionally, ongoing training and support for school staff are essential for the long-term success of these interventions. Continuous professional development for teachers and staff ensures that they have the knowledge and skills needed to effectively implement and sustain health programs. This includes training on the latest nutritional guidelines and PA recommendations [37].

Factors influencing effectiveness

Role of Family Involvement

**Figure 1:**
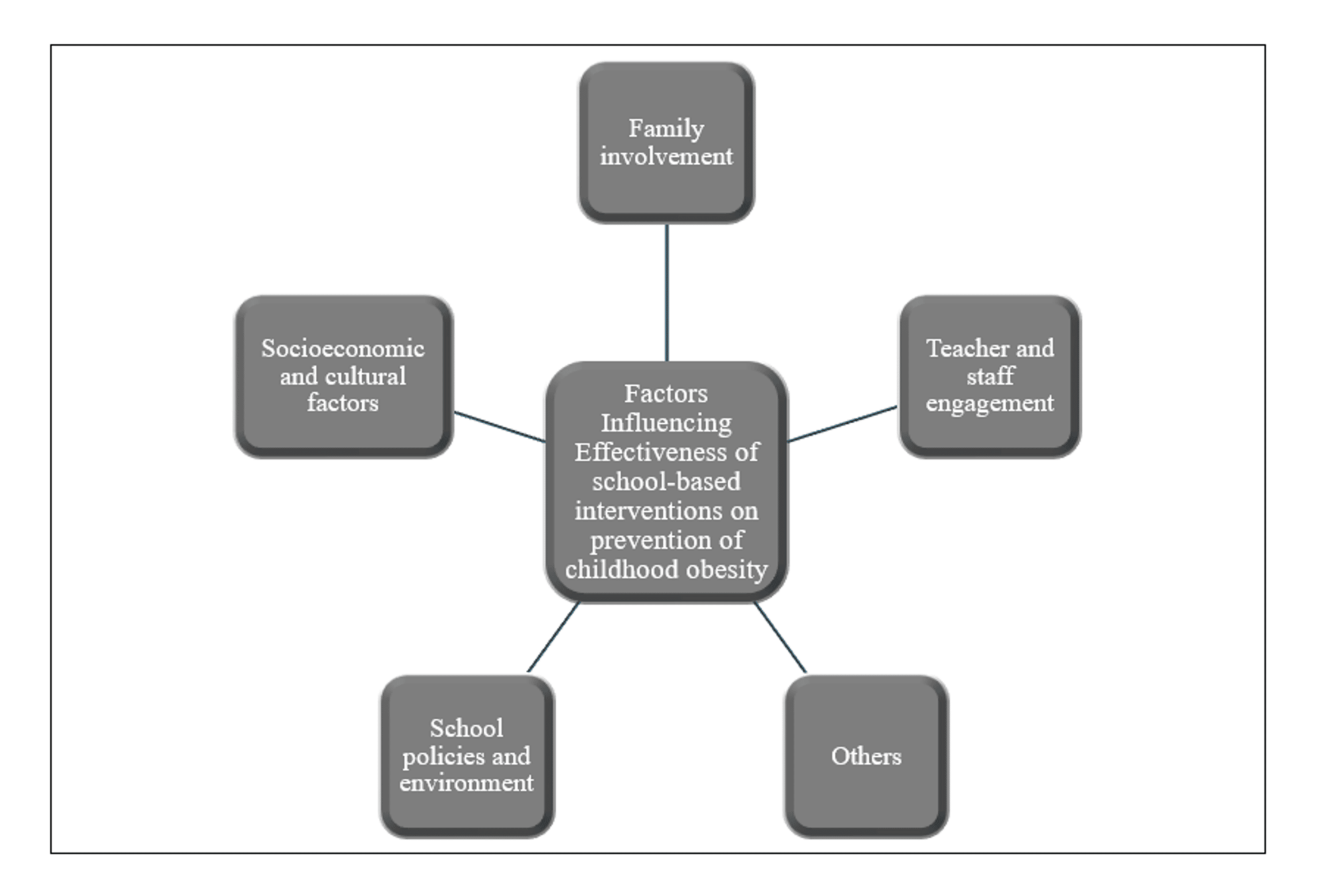
Factors influencing effectiveness of school-based interventions on prevention of childhood obesity. Image credit: All authors.

Several key factors influence school-based interventions designed to prevent obesity in children (Figure 1). Recent studies have shown that incorporating parents and caregivers in these interventions significantly enhances their impact. Family-based approaches provide a supportive environment that reinforces healthy behaviors learned at school, making it easier for children to adopt and maintain these behaviors especially before bad habits are established and before peer pressure influences dietary and activity choices in/out of school. Homs et al. showed the positive outcomes of family-based interventions in preventing obesity among school-age children, particularly in low socioeconomic status countries [38]. The study emphasized that involving parents in health-promotion activities, such as nutritional education and PA, led to significant improvements in children’s weight status and overall health behaviors. Similarly, other reviews highlighted effective strategies for childhood obesity prevention including school environment, stakeholder engagement, cultural relevance, policy support, training, funding, and monitoring, and found that school-based interventions with active family involvement were more successful in achieving favorable outcomes [39,40]. Children’s daily caloric and water needs vary by age, sex, weight, activity level, and growth stage, with recommended caloric intake ranging from 1,000 to 1,400 kcal/day for ages two to three years and increasing to 1,800-2,200 kcal/day by ages 11 to 12, while fluid needs can be calculated using weight-based formulas like 100 mL/kg for the first 10 kg and adjusted upward, emphasizing the importance of balanced nutrition and hydration for growth and health. A balanced diet should include proteins such as lean meats, fish, eggs, beans, and dairy for tissue repair and growth; carbohydrates from whole grains like brown rice, quinoa, and oats as a primary energy source; healthy fats from nuts, seeds, avocados, and oils like olive oil for brain development and energy; fruits and vegetables for essential vitamins, minerals, and fiber; and dairy or fortified plant-based alternatives for calcium and vitamin D to support bone health [41].

Teacher and Staff Engagement

Teacher and staff engagement is crucial in the effectiveness of school-based interventions, including structured training in intervention procedures (effective classroom management) to prevent childhood obesity. Active involvement of teachers and staff in intervention programs significantly enhances their success. Teachers’ engagement in training sessions and sustained implementation of intervention skills were associated with reduced disruptive behavior in students [42]. Similarly, another systematic review emphasized that teachers acting as role models and being actively involved in delivering interventions were effective in obesity prevention programs [43]. Other studies noted that interventions integrated into daily school practices and culture, with full staff engagement, yielded the most successful outcomes [39-44].

Socioeconomic and Cultural Factors

Children from lower socioeconomic backgrounds face a higher risk of obesity because they have limited access to healthy foods and safe recreational areas [40]. Socioeconomic status, race, and parental behaviors, such as smoking and family meal patterns, were strongly associated with childhood obesity [45]. Additionally, cultural factors, including dietary habits and PA levels, vary significantly across different communities, impacting the success of obesity prevention programs [46]. Qu et al. highlighted that Hispanic ethnicity and passive exposure to smoking were positively associated with childhood obesity, while higher household income and PA levels were protective factors. These findings show the need for tailored interventions that consider the unique socioeconomic and cultural contexts of the target populations to enhance their effectiveness [46]. Qu et al. revealed that children and adolescents born and raised in Mexico were less likely to be overweight or obese than their Mexican-American counterparts. Additionally, the prevalence of obesity and severe obesity was significantly lower among non-Hispanic White youth compared to non-Hispanic Black and other Hispanic youth [46].

School Policies and Environment

Policies promoting healthy eating and PA within schools can significantly impact children’s weight status and eating behaviors [46]. School policies restricting unhealthy food access and promoting healthy food choices were associated with lower BMI and reduced obesity risk among children [40,47]. Additionally, modifying the school environment, such as improving PA facilities and providing healthier food options, contributed to a decrease in obesity rates among school-aged children in Latin America [48]. In addition, Increasing PA time in weekly school schedules is crucial for enhancing student health, improving cognitive performance, fostering social skills, relieving stress, and promoting lifelong fitness habits [48].

Case studies and examples

Successful Intervention Programs 

Successful programs from various countries provide valuable insights into effective strategies for preventing childhood obesity. These strategies include policy changes, staff training, student engagement, healthier school meals, increased PA, and educational sessions on nutrition. These comprehensive approaches have led to significant improvements in children's dietary behaviors, PA levels, and fitness, and reductions in obesity rates. A summary of some examples are in Table 1.

**Table 1:** Examples of successful intervention programs of school-based interventions for preventing obesity in children.

Program name	Author and year	Country	Content of the program	Results of the program	Reference
Healthy Schools Program	Schwartz et al. (2015)	United States	Emphasized policy changes, staff training, and student engagement. Included healthier school meals, increased PA, and educational sessions on nutrition.	Notable decrease in obesity rates	[49]
Live Life Well at School	Hardy et al. (2015)	Australia	Aimed to increase PA levels and promote healthy eating through daily PA sessions and improved access to healthy foods.	Notable increase in students’ PA levels and a decrease in obesity rates	[50]
Daily Mile	Chesham et al. (2018)	United Kingdom	Encouraged children to run or jog for 15 minutes every day.	Improvements in children’s fitness levels and reductions in body fat	[51]
ToyBox	Manios et al. (2014)	Various European countries	Targeted preschool children and their families to promote healthy eating and PA. Included educational materials, interactive activities, and parental involvement.	Significant improvements in children’s dietary behaviors and PA levels	[52]

Lessons Learned From Less Effective Programs

Programs that concentrated solely on providing educational sessions and materials to parents, without advocating for necessary environmental and policy changes, were found to be less effective [43]. For example, interventions that did not include changes in the school environment, such as increasing access to healthy foods and PA programs, often failed to achieve significant outcomes [52,53]. Additionally, a lack of environmental and cultural support has been suggested to decrease their effectiveness in specific population groups [43]. 

A systematic review by Yuksel et al. emphasized that interventions with insufficient duration and intensity, as well as those lacking comprehensive strategies involving the whole school community, were less successful [53]. These findings underscore the importance of adding environmental, policy, and cultural considerations into school-based obesity prevention programs to enhance their effectiveness.

Challenges and limitations

One significant limitation is the lack of resources, including funding, time, and trained personnel [40]. Schools often struggle to allocate sufficient time within the curriculum for PA and nutrition education [54]. Additionally, there is often a lack of support from school staff and parents, which can hinder the successful implementation of these programs [55]. Cultural and socioeconomic factors also play a role, as interventions may not be tailored to the specific needs of diverse student populations [53].

The effectiveness of school-based interventions is often difficult to assess due to the differences in study designs and outcomes. Studies differ in their methodologies, duration, and components of the interventions, making it challenging to compare results and make definitive conclusions [56-58]. For instance, some programs focus only on PA, while others include dietary education or a combination of both. This variability can lead to inconsistent findings regarding the effect on BMI and outcomes [54,57].

Current research on school-based obesity prevention programs often lacks long-term follow-up, which is important for understanding the sustained impact of these interventions [59]. Many studies also have small sample sizes and are limited to specific geographic regions, reducing the generalizability of the findings. Furthermore, there is a need for more accurate evaluation methods to measure the effectiveness of these programs [60]. The reliance on self-reported data for dietary and PA behaviors can also introduce bias and affect the reliability of the results [56].

Future directions and recommendations

Future research should prioritize long-term follow-up studies to evaluate the sustained impact of school-based interventions on childhood obesity. Also, there is a need for more randomized controlled trials that include diverse populations to enhance the generalizability of the recommendations. Researchers should also study the use of technology, such as mobile apps to monitor and promote healthy behaviors among children [55,56].

Incorporating culturally tailored components can improve the effectiveness of interventions for diverse student populations. Schools should also consider involving parents and the broader community to create a supportive environment for healthy behaviors. Enhancing the training and involvement of school staff in delivering these programs can further improve their effectiveness [43,54].

Policymakers should prioritize funding for school-based obesity prevention programs and ensure that schools have the necessary resources to implement these interventions effectively. Policies should also support the integration of health education into the school curriculum and promote the availability of healthy food options in schools [60,61].

## Conclusions

School-based interventions that combine nutritional education and PA programs are effective in reducing BMI and improving weight status among children. Systematic reviews and extensive studies have demonstrated significant reductions in BMI and BMI z-scores through these comprehensive approaches. Integrating nutritional education into the school curriculum, including practical lessons and healthy food promotions, has shown positive impacts on dietary behaviors. 

Physical activity programs, such as regular physical education classes and extracurricular sports, are crucial in promoting healthy weight status. Parental involvement further enhances the effectiveness of these interventions, leading to better outcomes in weight status and energy balance-related behaviors. Overall, these multifaceted programs are essential in combating childhood obesity and promoting long-term health.
